# Stability, Intracellular Delivery, and Release of siRNA from Chitosan Nanoparticles Using Different Cross-Linkers

**DOI:** 10.1371/journal.pone.0128963

**Published:** 2015-06-11

**Authors:** Maria Abdul Ghafoor Raja, Haliza Katas, Thum Jing Wen

**Affiliations:** Centre for Drug Delivery Research, Faculty of Pharmacy, Universiti Kebangsaan Malaysia, Jalan Raja Muda Abdul Aziz, Kuala Lumpur, Malaysia; Universidad de Castilla-La Mancha, SPAIN

## Abstract

Chitosan (CS) nanoparticles have been extensively studied for siRNA delivery; however, their stability and efficacy are highly dependent on the types of cross-linker used. To address this issue, three common cross-linkers; tripolyphosphate (TPP), dextran sulphate (DS) and poly-D-glutamic acid (PGA) were used to prepare siRNA loaded CS-TPP/DS/PGA nanoparticles by ionic gelation method. The resulting nanoparticles were compared with regard to their physicochemical properties including particle size, zeta potential, morphology, binding and encapsulation efficiencies. Among all the formulations prepared with different cross linkers, CS-TPP-siRNA had the smallest particle size (ranged from 127 ± 9.7 to 455 ± 12.9 nm) with zeta potential ranged from +25.1 ± 1.5 to +39.4 ± 0.5 mV, and high entrapment (>95%) and binding efficiencies. Similarly, CS-TPP nanoparticles showed better siRNA protection during storage at 4˚C and as determined by serum protection assay. TEM micrographs revealed the assorted morphology of CS-TPP-siRNA nanoparticles in contrast to irregular morphology displayed by CS-DS-siRNA and CS-PGA-siRNA nanoparticles. All siRNA loaded CS-TPP/DS/PGA nanoparticles showed initial burst release followed by sustained release of siRNA. Moreover, all the formulations showed low and concentration-dependent cytotoxicity with human colorectal cancer cells (DLD-1), *in vitro*. The cellular uptake studies with CS-TPP-siRNA nanoparticles showed successful delivery of siRNA within cytoplasm of DLD-1 cells. The results demonstrate that ionically cross-linked CS-TPP nanoparticles are biocompatible non-viral gene delivery system and generate a solid ground for further optimization studies, for example with regard to steric stabilization and targeting.

## Introduction

Small interfering RNAs (siRNAs) have the potential for therapeutic application in many diseases, including cancer [1,2]. Despite efficient and reliable gene silencing activity *in vitro*, only limited siRNA intracellular delivery has been achieved, owing to its rapid enzymatic degradation and poor cellular uptake [3,4]. Therefore, effective delivery systems that can protect siRNAs and transport them into the cytoplasm of target cells are needed to exploit the full therapeutic potential of siRNAs [5].

Viral and non-viral vectors are used as carriers to deliver genes. Although viral vectors have higher transfection efficiencies than non-viral vectors in most cells, safety concerns have been raised in numerous clinical trials[6]. Non-viral vectors have garnered the focus of researchers because of their ease of synthesis and modification, low immunogenicity, and controllable size[7]. Non-viral delivery systems using cationic liposomes and polymers, such as polyethylenimine (PEI), poly (l-lysine) (PLL), and their respective derivatives have been used to condense siRNAs to form nanoparticles[8,9]. However, liposomal-based formulations are often toxic to cells and are quickly cleared from the bloodstream[10,11]. Among the recent materials studied for use in polymeric nanoparticle synthesis, chitosan (CS) has been favoured as a potential nanoparticle carrier owing to its unique properties[12–14]. CS is a linear polysaccharide composed of glucosamine and *N*-acetyl glucosamine residues, and can be derived by partial deacetylation of chitin[15]. CS is known to be biocompatible, minimally toxic, nonimmunogenic, and degradable by enzymes[16–19]. Despite these advantages, the stability of CS nanoparticles remains a crucial issue for researchers.

Cross-linkers play an important role in the preparation of stable nanoparticles. The most widely used cross-linker in the preparation of CS nanoparticles is sodium tripolyphosphate (TPP), which is a small, anionic, and non-toxic cross-linking agent [20]. The primary limitation of TPP is that is has limited sites for ionotropic gelation[21]. Another polyanion commonly used to cross-link CS is dextran sulfate (DS). DS is used in pharmaceutical fields because of its biodegradability and biocompatibility[22]. Furthermore, DS is a highly branched polysaccharide with glycosidic linkages that contribute approximately 2.3 sulfate groups per glucosyl unit[22]. Recently, studies have been carried out on poly-d-glutamic acid sodium salt (PGA) as an anion in the formation of CS nanoparticles. PGA is a natural water-soluble anionic polypeptide that is biodegradable and edible, and has no reported toxicity[23]. Because of its good tissue affinity, it has attracted researchers’ interest[24].

Although, the effect of TPP and PGA was previously compared for CS nanoparticles, the particles however were loaded with BSA[25]. Besides that, the storage and serum stability and cytotoxicity effects of CS nanoparticles were not comprehensively investigated. As the success of a drug delivery system is highly dependent on its physical characteristics and stability, the objective of this study was to look into the effects of different cross-linkers not only from the aspect of physical characteristics but also stability of siRNA-loaded CS nanoparticles. In addition, present study provided a comparison of cytotoxicity effects and cellular internalization of siRNA-loaded CS nanoparticles cross linked by TPP, DS and PGA. The findings were expected to aid in the selection of the most appropriate crosslinker to develop a safe, stable and efficient gene delivery system.

## Materials and Methods

### 2.1 Materials

Low-molecular-weight (192 kDa) CS with a 75–85% degree of deacetylation (DD) was obtained from Sigma-Aldrich (St. Louis, MO, USA), penta-sodium tripolyphosphate (TPP) was obtained from Merck (Darmstadt, Germany), DS sodium salt (DS)(MW = 500 kDa) was obtained from Fisher Scientific (Ontario, Canada), and (PGA)(MW = 15–50 kDa) was obtained from Sigma-Aldrich (St. Louis, MO, USA). siRNAs of 21 base pairs targeting the *VEGF* gene [GGAGUACCCUGAUGAGAUCdtdt] and Hoechsct 33342 stain were obtained from Thermo Scientific Dharmacon (Lafayette, CO, USA). Live/dead cell viability assay kits, alamarBlue reagent, and a 10-bp DNA ladder were obtained from Invitrogen (Carlsbad, CA, USA). Human colorectal adenocarcinoma cells (DLD-1) were obtained from ATCC (Manassas, VA, USA). Phosphate-buffered saline (PBS) was obtained from Sigma-Aldrich (St. Louis, MO, USA). Dulbecco’s modified Eagle’s medium (DMEM), penicillin-streptomycin (penstrep), and fetal bovine serum (FBS) were purchased from Gibco (New York, USA). Heparin sodium was purchased from Leo (Ballerup, Denmark). Deionized water of resistivity 18.2MΩcm was used in all the experiments. Acetic acid and other chemicals were of analytical grade.

### 2.2 Preparation of CS nanoparticles

#### 2.2.1 Ionic gelation

CS nanoparticles were prepared via ionic gelation methods established by Calvo et al. with some modifications[26]. CS solutions (0.1%, 0.2%, 0.3%, and 0.4% w/v) were prepared by dissolving CS in 2% v/v glacial acetic acid. Three cross-linking agents (TPP, DS, and PGA) were investigated. TPP solution (0.1% w/v) was prepared by dissolving TPP in deionized water, and DS and PGA solutions were prepared using the same method. CS nanoparticles were prepared by adding 1.2 mL of cross-linker aqueous solution dropwise into 3 mL of CS solutions (0.1%, 0.2%, 0.3%, and 0.4% w/v) at room temperature, with constant magnetic stirring (MS MP8 Wise Stir Wertheim, Germany) at 700 rpm for 30 min. The nanoparticles were later incubated for another 30 min at room temperature before further analysis. Centrifugation (Optima L-100 XP Ultracentrifuge, Beckman-Coulter, CA, USA) was performed at 13,000 x g at 10°C for 30 min to collect nanoparticles. The supernatants were discarded and pellets of nanoparticles were re-suspended in filtered (0.25-μm Millex GP filter unit, Millipore, Billerica, MA) deionized water.

#### 2.2.2 siRNA entrapment

To associate siRNAs with the CS-TPP, CS-DS, and CS-PGA nanoparticles, 3μL of siRNAs (19 μg/μL) was added to 1.2 mL of cross-linker in aqueous solution (0.1% w/v), and this was added to 3 mL of CS solution (0.1%, 0.2%, 0.3%, and 0.4% w/v) under constant magnetic stirring (700 rpm) at room temperature. The particles were then incubated at room temperature for another 30 min before further analysis.

### 2.3 *In Vitro* Characterization of CS-TPP/DS/PGA siRNA nanoparticles

#### 2.3.1 Particle size, PDI, zeta potential

The mean particle diameter (z-average), polydispersity (PDI), and zeta potential (surface charge) of freshly prepared and CS nanoparticles were determined by photon correlation spectroscopy (PCS) using ZS-90 Zetasizer (Malvern Instruments, Worcestershire, UK). All measurements were performed after the samples were harvested by centrifugation and re-suspended in deionized distilled water. Each sample was assayed in triplicate at 25°C, and data are reported as mean±standard deviation.

#### 2.3.2 Morphological analysis

Morphological characterization of unloaded and siRNA-loaded CS-TPP/DS/PGA nanoparticles was carried out using transmission electron microscopy (TEM)(Tecnai Spirit, FEI, Eindhoven,The Netherlands). A drop of nanoparticles dispersion was placed on the copper microgrid that was natively stained by 3% w/v phosphotungstic acid. The stained nanoparticles was incubated for 5–10 min and evaporated at room temperature (25 ± 2°C). Then, it was viewed under the TEM for imaging of samples.

#### 2.3.3 Entrapment efficiency

The entrapment efficiency of siRNAs (% entrapped) for CS-TPP/DS/PGA nanoparticles was measured using a UV-vis spectrophotometer (Shimadzu UV-1800, Shimadzu Scientific Instruments, Japan) at 260 nm. Briefly, the free siRNA in supernatant recovered from centrifugation (13,000 x g at 10°C for 30 min) was quantified by measuring its absorbance at 260 nm wavelength (the maximum absorption of nitrogenous bases of nucleotides) with a dual beam UV-vis spectrophotometer. Concentration of free siRNA was determined using Beer’s Law (A_260_ ƐCL) where C is the concentration of siRNA, A_260_ is the absorbance at 260 nm, Ɛ is the extinction coefficient and L is the path length of the cuvette. Extinction coefficient of siRNA is 385101Lmol^-1^cm^-1^ and entrapment efficiency was calculated using the following formula:

$$\text{Entrapment efficiency}\left(\text{\%}\right)=\frac{\text{Csample}-\text{Csupernatant}}{\text{Csample}}\times 100$$ 1

where C_sample_ is the concentration of siRNA added and C_supernatant_ is the concentration of siRNA in the supernatant. All measurements were performed in triplicate, and data are reported as mean ± standard deviation.

#### 2.3.4 Gel retardation assay

The binding efficiency of siRNA to CS-TPP/DS/PGA nanoparticles was determined using 4% w/v agarose gel electrophoresis and SYBR Green (Invitrogen, Carlsbad, CA, USA) staining. Twenty microliters of sample (prepared at various CS concentrations) containing 0.2 μg of siRNA was loaded into the wells. A 10-bp DNA ladder was used as a size reference. Free siRNA and unloaded CS nanoparticles were used as positive and negative controls, respectively. The siRNA bands were viewed using a real-time UV transilluminator at 480 nm as per manufacturer’s protocol (Invitrogen, Carlsbad, CA, USA). Moreover, the quantitative analysis was performed by using imageJ software (version 1.45s) developed by National Institutes of Health (NIH, USA).

### 2.4 Stability studies

#### 2.4.1 Serum protection assay

siRNA-loaded CS-TPP/DS/PGA nanoparticles prepared from 0.1% w/v CS were selected for serum protection assay because of their small particle size, net positive charge, and high entrapment efficiency. A volume of 200 μL of siRNA-loaded CS nanoparticles (containing 5 μg of siRNA) was incubated at 37°C with an equal volume of RPMI supplemented with 10% FBS. Naked siRNA acted as a control and was treated in the same manner. At each time interval (0 min, 30 min, 2 h, 4 h, 24 h, and 48 h), 40 μL of the mixture was removed and stored at -20°C in preparation for gel electrophoresis. Free siRNA was used as an internal control. Before performing gel electrophoresis, the samples were incubated in a bath incubator at 60°C for 3 min to terminate serum activity. A volume of 5 μL of heparin (1000 U/mL) was then added to displace siRNA from the CS-TPP/DS/PGA nanoparticles. The integrity of the siRNA displaced from nanoparticles was analyzed by conducting gel electrophoresis with 4% w/v agarose gel stained with SYBR Green (Invitrogen, Carlsbad, CA, USA). Electrophoresis was performed for 30 min at 110 volts and siRNA bands were visualized under a real-time UV transilluminator at 480 nm (Invitrogen, Carlsbad, CA, USA). Moreover, the quantitative analysis was performed by using imageJ software developed by National Institutes of Health (NIH).

#### 2.4.2 Storage stability of CS nanoparticles

CS concentration of 0.1% w/v was used to prepare CS nanoparticles for this and further experiments. CS-TPP/DS/PGA nanoparticles loaded with siRNA were suspended in deionized distilled water andin PBS (pH 7.4), stored at 4°C for 15 days.The storage stability of nanoparticles was determined by measuring the particle size of CS nanoparticles at pre-determined time points.

### 2.5 In vitro release studies

The release characteristics of siRNA were studied using PBS (pH 7.4). Samples (4 mL) were centrifuged at 13,000 x g for 30 min at 37°C and pellets were re-suspended in 3 mL of PBS. The re-suspended pellets were stirred at 100 rpm by using a magnetic stirrer for 8 days at 37°C. At different time intervals, the samples were centrifuged at 13,000 x g for 30 min at 25°C. The supernatant recovered from centrifugation was used for further analysis, and an equivalent volume of fresh PBS was added to the sample. The amount of siRNA present in the supernatant was analyzed using a UV-vis spectrophotometer (Shimadzu UV-1800, Shimadzu Scientific Instruments, Japan) at 260 nm.

### 2.6 Cytotoxicity studies

DLD-1 cells (ATCC, Manassas, VA, USA) were cultured in RPMI 1640 medium at a cell density of 4 × 10^4^ per well. The cells were supplemented with a medium containing 10% FBS and 1% penstrep, and maintained at 37°C in a humidified 5% CO_2_/95% air atmosphere. After 24 h and 48 h of incubation of untreated cells, naked siRNA and siRNA-loaded CS-TPP/DS/PGA nanoparticles at 37°C, a final dilution of 1/10 per cell volume of alamarBlue reagent was added to the treated cells, followed by incubation for 4 h prior to analysis. The absorbance of each sample at 570 nm (A570, the maximum absorption of alamarBlue product (resorufin)) was measured on a microplate reader (Varioskan Flash, Thermo Scientific, Waltham, MA, USA). The assay was performed in the presence of 10% FBS. Cell viability was determined using the following equation:

$$Cellviability\left(\%\right)=\frac{\text{A}570\;\text{of treated cells}}{\text{A}570\;\text{of control cells}}\times 100$$ 2

The amount of FBS proteins in the supernatant recovered after ultracentrifugation was determined via Bradford Protein Assay Kit (Sigma Aldrich St. Louis, MO, USA). The FBS protein adsorption efficiency was calculated by the following equation:

$$\text{Protein adsorption}\left(\text{\%}\right)=\frac{\text{Ctotal}-\text{Csup}}{\text{Ctotal}}\times 100$$ 3

where C_total_ represents the total amount of protein in medium and C_sup_ is the concentration of protein in supernatant.

#### 2.6.1 LIVE/DEAD cell viability assay

This assay was performed to measure the functional status of the cell by detecting cytoplasmic esterase activity using the LIVE/DEAD Viability/Cytotoxicity kit for mammalian cells (Invitrogen, Carlsbad, CA, USA), which contains calcein, which fluoresces green in living cells, and ethidium bromide, which fluoresces red in dead cells. This assay was performed in 96-well plates. Briefly, DLD-1 cells were plated at a seeding density of 2× 10^4^ per well. The cells were supplemented with a medium containing 10% FBS and 1% penstrep, and maintained at 37°C in a humidified 5% CO_2_/95% air atmosphere. The cells were treated with siRNA-loaded CS-TPP/DS/PGA nanoparticles for 24 h and 48 h. The assay was performed in the presence of 10% FBS. Subsequently, the cells were rinsed twice with PBS, fluorochromes (calcein/ethidium bromide) were added, and cells were incubated for 45 min. Finally, reagents were removed and cells were analyzed with a Floid Cell Imaging Station (Molecular Probes Life Technology, France) for calcein and ethidium bromide flourescence.

### 2.7 Cellular internalization

To determine successful siRNA internalization, DLD-1 cells (3×10^4^) were seeded in a 96-well tissue culture plate and cultured for 24 h (80% confluence). The cells in each well were incubated for 4 h in a medium with 10% FBS with free 6-FAM-siRNA, or with siRNA carried by CS-TPP nanoparticles. After incubation, the cells were washed twice with PBS and then stained with 1μg/mL of Hoechst stain 33342 for 15 min at 37°C. Studies were performed using a Floid Cell Imaging Station (Molecular Probes Life Technology, France).

### 2.8 Statistical analysis

Data are presented as mean ± standard deviation (SD), and were analyzed by 1-way ANOVA with Tukey post-hoc tests using SPSS 21.0. *p*-values <0.05 indicated statistical significance of differences between groups.

## Results and Discussion

### 3.1 Particle size, PDI, and zeta potential

The mean particle size of siRNA-loaded CS-TPP/DS/PGA nanoparticles was increased significantly by increasing the CS concentration from 0.1% to 0.4% w/v, as shown in Table 1. This was expected because of the lower viscosity observed at lower concentrations of CS, which resulted in better solubility and interaction between CS and crosslinkers, and thus smaller particle size[27]. Moreover, significant decrease in particle size was observed after loading of siRNA into CS-TPP/DS/PGA nanoparticles in comparison to unloaded CS-TPP/DS/PGA nanoparticles as shown in (Table 2), which was in accordance with previous findings[28]. Among TPP/DS/PGA, CS-TPP-siRNA nanoparticles had the smallest particle size range as compared to the nanoparticles obtained from DS and PGA, as shown in Table 1.TPP produces small particles because it is a small polyanionic molecule that forms strong ionic interactions with the NH_3_^+^ groups of CS[27].

**Table 1:** Particle size, PDI, and zeta potential of siRNA-loaded CS-TPP/DS/PG Ananoparticles prepared at different CS concentrations, *n* = 3.

CS concentration	Particle size		Zeta potential
(% w/v)	(nm) ± SD	PDI ± SD	(mV) ± SD
**CS-TPP-siRNA**			
0.1	127.4 ± 10.7	0.3 ± 0.13	+25.1 ± 1.5
0.2	223.1 ± 16.7	0.4 ± 0.19	+30.7 ± 1.1
0.3	295.1 ± 29.6	0.4 ± 0.13	+35.5 ± 1.0
0.4	455.3 ± 12.9	0.5 ± 0.18	+39.4 ± 0.5
**CS-DS-siRNA**			
0.1	829.4 ± 10.0	0.6 ± 0.08	+71.9 ± 1.8
0.2	976.2 ±± 70.8	0.6 ± 0.19	+74.5 ± 0.3
0.3	1203.3 ± 37.7	0.9 ± 0.09	+76.2 ± 0.8
0.4	1682.0 ± 56.4	1.0 ± 0.26	+79.6 ± 0.6
**CS-PGA-siRNA**			
0.1	497.9 ± 55.6	0.5 ± 0.08	+62.9 ± 2.3
0.2	959.6 ± 16.0	0.7 ± 0.10	+69.8 ± 2.2
0.3	1091.3 ± 21.5	0.9 ± 0.00	+72.3 ± 1.0
0.4	1739.3 ± 71.2	1.0 ± 0.09	+77.7 ± 0.5

**Table 2:** Particle size, PDI, and zeta potential of unloaded CS-TPP/DS/PG Ananoparticles prepared at different CS concentrations, *n* = 3.

CS concentration	Particle size	PDI ± SD	Zeta potential
(% w/v)	(nm) ± SD		(mV) ± SD
**CS-TPP nanoparticles**			
0.1	179.9 ± 3.9	0.3 ± 0.10	+30.1 ± 1.0
0.2	272.3 ± 9.0	0.3 ± 0.29	+35.5 ± 0.5
0.3	342.0 ± 7.0	0.4 ± 0.03	+42.5 ± 1.0
0.4	506.0 ± 8.1	0.6 ± 0.08	+45.7 ± 0.5
**CS-DS nanoparticles**			
0.1	909.5 ± 12.9	0.6 ± 0.01	+74.7 ± 0.5
0.2	1151.7 ± 40.1	0.6 ± 0.20	+78.5 ± 1.0
0.3	1457.7 ± 10.0	0.8 ± 0.06	+78.2 ± 0.4
0.4	1882.0 ± 62.8	1.0 ± 0.05	+84.1 ± 0.7
**CS-PGA nanoparticles**			
0.1	980.9 ± 55.6	0.6 ± 0.09	+68.7 ± 1.0
0.2	1466.7±23.7	0.8 ± 0.10	+74.4 ± 0.9
0.3	1571.7±40.5	0.8 ± 0.05	+77.8 ± 1.2
0.4	1940.2 ±91.2	1.0 ± 0.09	+83.0 ± 2.0

**Fig 1:**
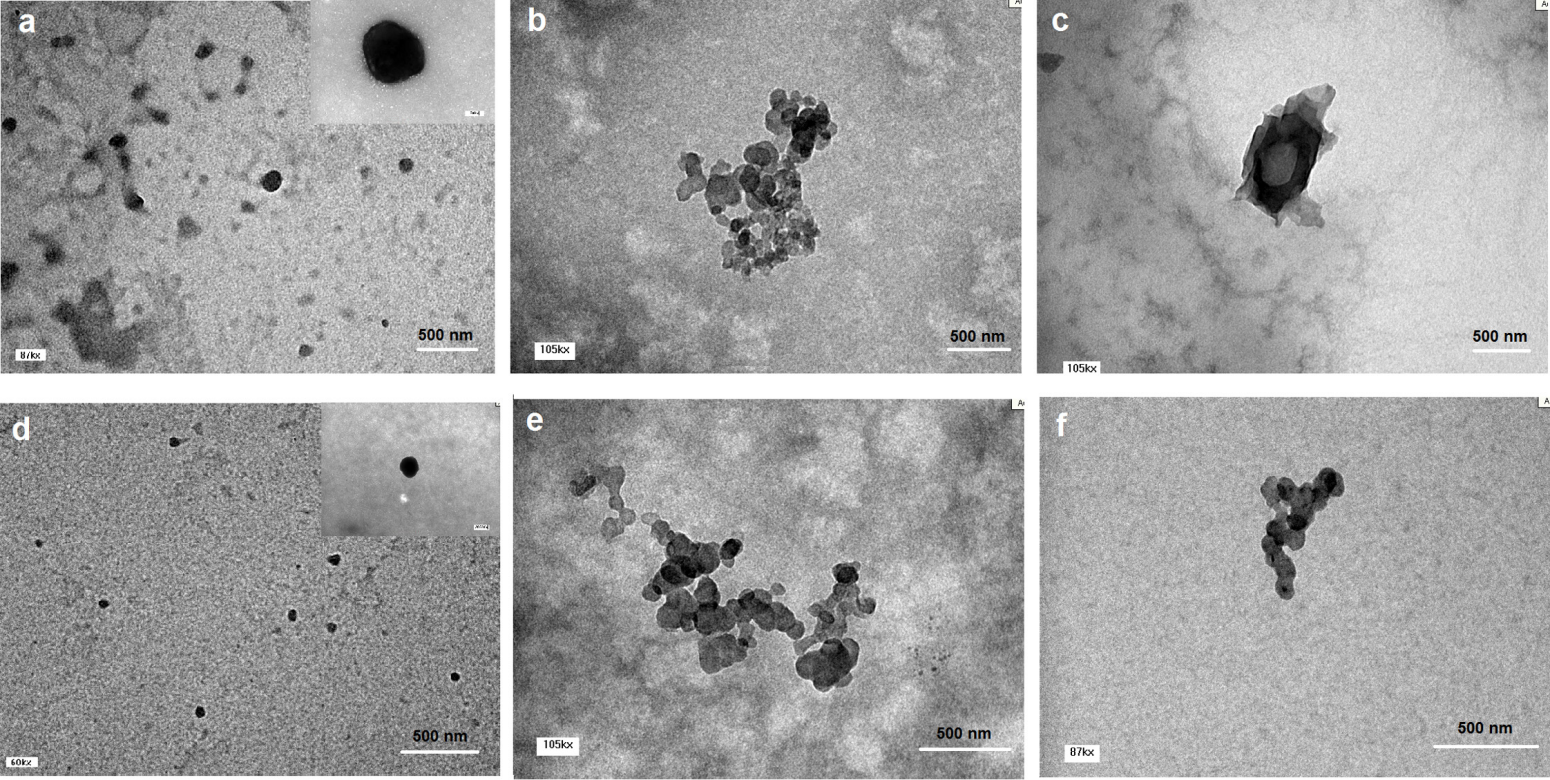
TEM images of CS nanoparticles (0.1% w/v CS). Blank CS-TPP nanoparticles (a), blank CS-DS nanoparticles (b), blank CS-PGA nanoparticles (c), CS-TPP-siRNA nanoparticles (d), CS-DS-siRNA nanoparticles (e), and CS-PGA-siRNA nanoparticles (f) at different magnifications (60 kx, 105 kx, 87 kx).

In contrast, CS-PGA-siRNA nanoparticles had significantly larger particle sizes across CS concentrations, as shown in Table 1, which could be due to the fact that PGA is a macromolecule with fewer ionic interactions with CS NH_3_^+^groups[25]. CS-DS-siRNA also showed a large particle size which could be due to the aggregation of small particles that tend to fuse together and form groups, as shown in Fig 1[29].

All cross-linker formulations showed PDI values from 0.2 to 1, and values increased as CS concentration increased from 0.1% to 0.4% w/v (Table 1). PDI values of formulations using TPP as the cross-linking agent were within the acceptable range, indicating narrow distribution of particle size, while PGA showed high PDI values, implying broad distribution of particle size.

The surface charge of unloaded CS nanoparticles ranged from approximately +30 to +85mV (Table 2) at CS concentrations from 0.1% to 0.4% w/v. In general, the zeta potential of siRNA-loaded CS-TPP/DS/PGA increased as the concentration of CS increased (Table 1), because a greater number of excess free positive charges were available that did not counteract the negatively charged siRNA (the amount of siRNA was fixed)[28]. Moreover, the decrease in zeta potential after siRNA loading demonstrated the succesful association of siRNA with the particles. The presence of siRNA phosphate groups would neutralize CS NH_3_^+^ groups, and therefore lower the positive charges of cationic CS[28,30]. TPP produced nanoparticles that exhibited low zeta potential, and DS and PGA produced nanoparticles with high zeta potential (Table 1). This result could be caused by the strong cross-linking between TPP and the NH_3_^+^ groups of CS, which would result in neutralization of NH_3_^+^ groups and low zeta potential. Higher zeta potential was observed for other cross-linkers because of the lower degree of cross-linking between PGA/DS and CS[30].

### 3.2 Morphology

Images of CS nanoparticles loaded with siRNA obtained by TEM are shown in Fig 1. Fig 1(A) shows an assorted morphology of unloaded CS-TPP nanoparticles, consisting mostly spherical and some irregular particles. Similar morphology was observed when nanoparticles were loaded with siRNA as shown in Fig 1(D). However, unloaded CS-DS/PGA nanoparticles showed aggregation and irregular morphology (Fig 1B and 1C). Similarly, CS-DS-siRNA and CS-PGA-siRNA showed aggregation due to inefficient cross-linking (Fig 1E and 1F).

Variation in cross-linking ability was expected to contribute to differences in morphology. TPP showed excellent cross-linking ability and successfully neutralized CSNH_3_^+^groups, resulting in small and more spherical nanoparticles. In contrast, DS and PGA did not cross-link CS as efficiently as TPP at the concentrations studied, which resulted in irregular morphology. Moreover, aggregation was most likely due to lower degree of cross linking between DS or PGA and CS [30], resulted in excess positive charge from the unneutralized CS NH_3_^+^ groups. It has been reported that under acidic conditions, electrostatic repulsion and interchain hydrogen bonding interactions exist in equilibrium when CS concentration below a certain limit. Above this limit, NH_3_^+^groups tend to cause surface shielding and result in domination of intermolecular hydrogen bonding during cross linking process. This leads to plenty of CS molecules involved in the cross-linking of a single particle and subsequently large particles are formed. The formation of large particles leads to flocculent precipitate because electrostatic repulsion between particles is not sufficient to maintain the stability of these large particles [27].

### 3.3 siRNA entrapment efficiency

**Fig 2:**
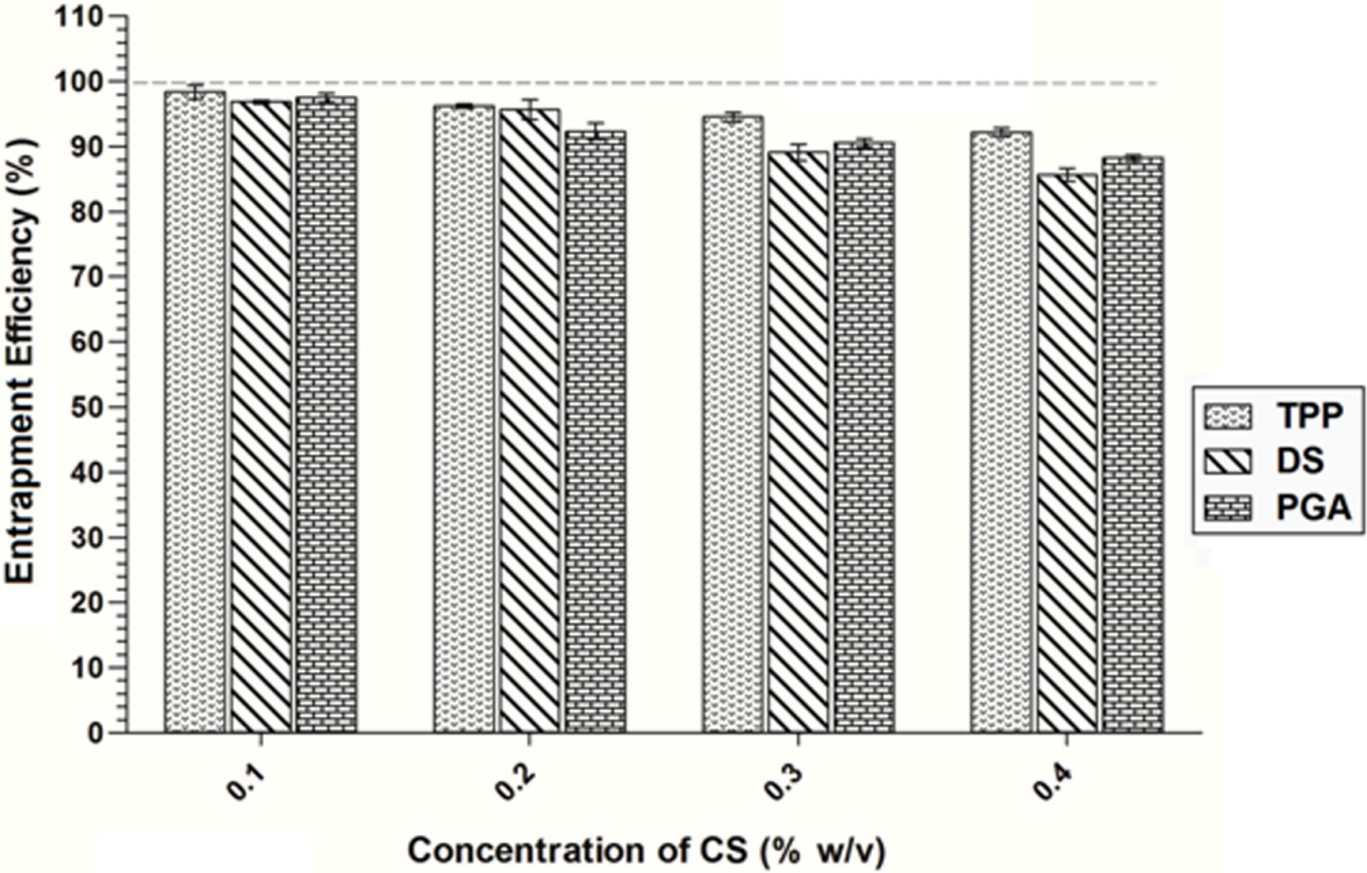
Entrapment efficiency of siRNA-loaded CS-TPP/DS/PGA nanoparticles prepared using different CS concentrations (0.1% to 0.4% w/v CS), *n* = 3.

An siRNA entrapment efficiency in the range of 85% to 99% was achieved for CS-TPP/DS/PGA nanoparticles. In general, the entrapment efficiencies of all formulations decreased when CS concentration was increased, as shown in Fig 2. The entrapment efficiency of siRNA-loaded CS-TPP, CS-DS, and CS-PGA formulations was decreased significantly by increasing CS concentration from 0.1% to 0.4% w/v. Higher CS concentrations produced a more viscous solution that hindered the movement of siRNA around the CS chain and led to inefficient siRNA entrapment[31]. The low entrapment efficiency of CS-DS-siRNA and CS-PGA-siRNA may be explained by shielding effects and steric hindrance, which interfered with the interaction between siRNA and CS NH_3_^+^ groups[32].

### 3.4 Binding efficiency of siRNA with CS nanoparticles

**Fig 3:**
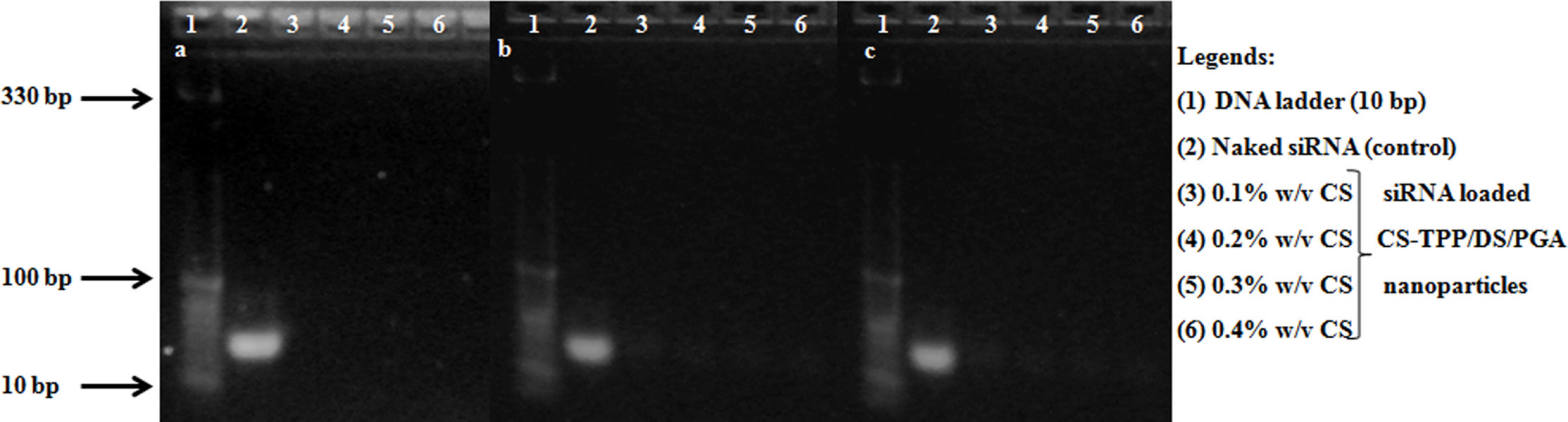
Binding efficiency of siRNA-loaded CS nanoparticles as determined by 4% w/v agarose gel electrophoresis. CS-TPP-siRNA (a), CS-DS-siRNA (b), and CS-PGA-siRNA nanoparticles (c).

To further investigate siRNA binding to CS-TPP/DS/PGA nanoparticles, agarose gel electrophoresis was performed. For CS-TPP-siRNA, complete binding of siRNA to CS nanoparticles was observed (due to the absence of a trailing band), which suggested a strong interaction occured between CS and siRNA, as shown in Fig 3 (A). However, CS-DS-siRNA and CS-PGA-siRNA showed trailing bands that could indicate some release of siRNA from nanoparticles as shown in Fig 3(B) and 3(C), respectively. The results also showed that the interaction between CS and siRNA in the presence of DS and PGA was comparatively weaker than in the presence of TPP. The findings were further supported by quantitative analysis of relative density of siRNA bands using imageJ software (Table 3).

**Table 3:** Quantification of binding efficiency assay of siRNA loaded CS-TPP/DS/PGA nanoparticles.

Concentration Of CS % (w/v)	Area	Percent	Relative density
**CS-TPP-siRNA**			
0.1	ND*	ND	ND
0.2	ND	ND	ND
0.3	ND	ND	ND
0.4	ND	ND	ND
**CS-DS-siRNA**			
0.1	2810.42	6.216	0.07
0.2	1643.79	3.466	0.04
0.3	1345.84	2.519	0.03
0.4	1125.25	1.688	0.02
**CS-PGA-siRNA**			
0.1	2865.06	7.418	0.09
0.2	1641.2	4.251	0.05
0.3	1390.25	3.601	0.04
0.4	1148.37	2.973	0.03

Mean percent of control is 80 ± 6 and relative density is 1.

* ND stands for no data as no bands detected

### 3.5 Stability studies

#### 3.5.1 Serum protection test

**Fig 4:**
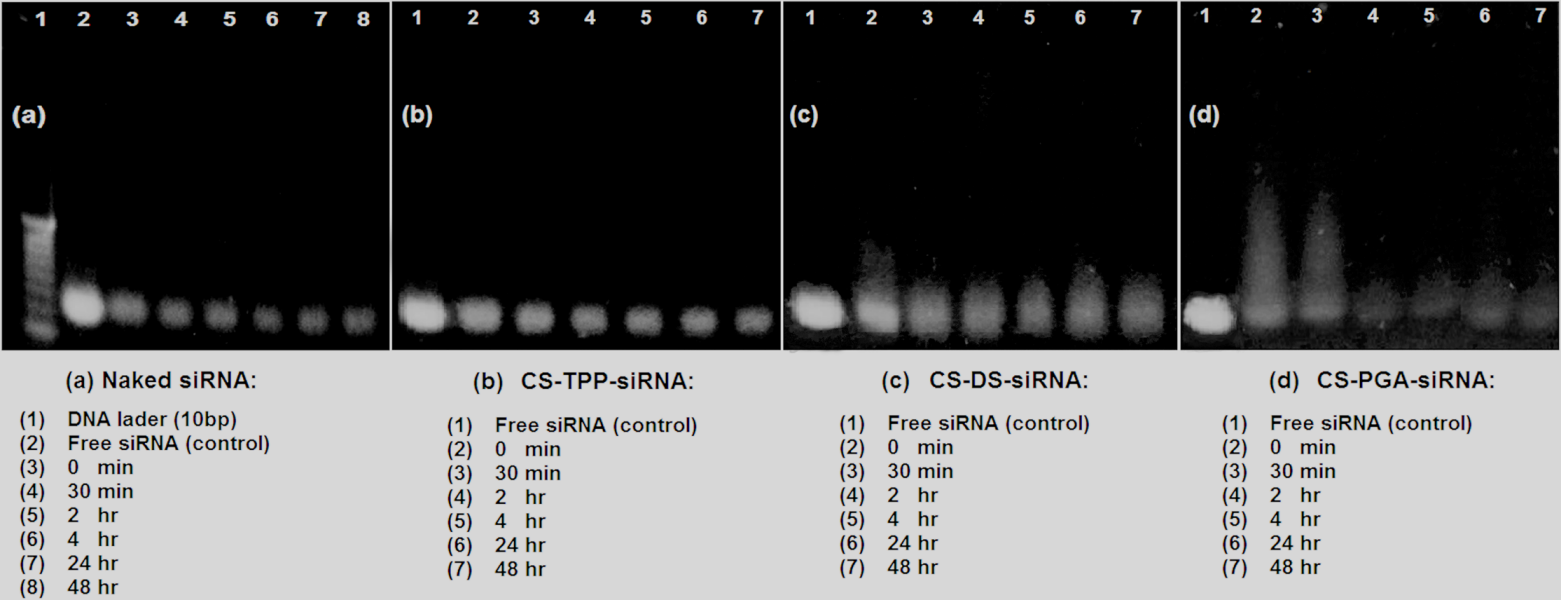
Electrophoretic mobility of naked siRNA (a), CS-TPP-siRNA (b), CS-DS-siRNA (c), CS-PGA-siRNA nanoparticles (d), following incubation in RPMI medium containing 10% FBS. Graph representing relative density (quantification) of gel electrophoresis bands of serum protection assay, by imageJ software (e).

The ability of a carrier to protect its payload from nuclease degradation is an important property for efficient gene delivery. siRNA must be protected fromnuclease digestion for maximal activity in the cells. To address this, serum protection test was carried out for CS nanoparticles in 10% FBS. Fig 4 shows trailing bands for siRNA-loaded CS-TPP/DS/PGA nanoparticles, indicating the migration of siRNA from nanoparticles after treatment with heparin. Thus, it was assumed that some siRNA had been unbound from nanoparticles and interacted with components in FBS. The interaction between unbound siRNA and components in FBS had contributed to the appearance of trailing bands due to slower migration of siRNA. The bands were not as intense as the non-treated free siRNA (control) because some siRNA might remain within the nanoparticles (could not be stained by SYBR Green) or be degraded. Naked siRNA started to degrade as early as 0 min with degradation resulting during the mixing of siRNA with serum and freezing steps [28]and most of thes iRNA was degraded after 48 h of incubation (Fig 4(A)). In comparison to naked siRNA, CS-TPP nanoparticles effectively protected siRNA from nuclease degradation up to 48h as brighter and dense band was observed (Fig 4(B)). In contrast, CS-DS and CS-PGA nanoparticles partially protected siRNAafter 48h as lesser dense band was observed compared to CS-TPP (Fig 4(C)). In case of CS-PGA nanoparticles, the least dense trailing bands indicate the probability of more siRNA was being degraded and interacted with FBS components. In order to support the findings, the quantitative analysis of bands of naked siRNA, siRNA-loaded into CS-TPP/DS/PGA nanoparticles was performed by using an ImageJ software (as shown in Table 4). The results showed CS-TPP-siRNA provide a better protection for siRNA than CS-DS and CS-PGA nanoparticles (Fig 4(E)). The relative density of siRNA loaded CS-TPP is approximately two-folds higher than CS-DS and three-folds higher than CS-PGA nanoparticles, after 48 h incubation in serum. Naked siRNA showed the least relative density compared to siRNA carried by CS nanoparticles. Thus, CS-TPP nanoparticles effectively protected siRNA from the enzymatic activity of serum components. However, CS-DS and CS-PGA nanoparticles provided partial protection for siRNA when compared with CS-TPP nanoparticles.

**Table 4:** Quantification of serum protection assay of siRNA loaded CS-TPP/DS/PGA nanoparticles.

Incubation time in serum (h)	Area	Percent	Relative density
**CS-TPP-siRNA**			
0	43408.74	28.775	0.622
0.5	30124.56	19.969	0.425
2	23145.16	15.343	0.331
4	19692.48	13.054	0.282
24	17265.93	11.445	0.247
48	17217.74	11.414	0.246
**CS-DS-siRNA**			
0	28629.84	17.648	0.381
0.5	11609.25	7.156	0.154
2	9410.43	5.801	0.125
4	9628.14	5.935	0.128
24	7414.3	4.999	0.108
48	7409.68	4.771	0.103
**CS-PGA-siRNA**			
0	18545.68	13.525	0.292
0.5	13619.47	9.932	0.214
2	13526.89	9.865	0.213
4	10055.4	7.333	0.158
24	7555.43	5.51	0.119
48	7328.2	3.835	0.082
**Naked siRNA**			
0	6557.51	0.091	0.091
0.5	3856.16	0.053	0.053
2	3844.74	0.053	0.053
4	1616.74	0.022	0.022
24	1330.33	0.017	0.017
48	1200.05	0.018	0.018

Relative density of control is 1 and percent is 46.26 ± 1.

#### 3.5.2 Storage stability of siRNA loaded CS nanoparticles

**Fig 5:**
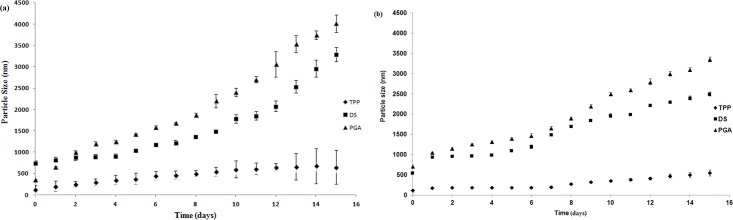
Storage stability of siRNA-loaded CS-TPP/DS/PGA nanoparticles in deionized distilled water (a), and PBS (b) at 4°C, *n* = 3.

Storage stability of siRNA-loaded CS-TPP/DS/PGA nanoparticles in deionized distilled water is shown in Fig 5(A).Deionized distilled water was used in order to determine nanoparticles’ stability in the medium in which they were suspended as reported by others [33,34]. In cases where the nanoparticles are stable in their suspending medium, additional step to stabilize the nanoparticles such as lyophilisation could be avoided [35]. The previous findings have indicated that cross-linking reactions improve the properties of particulates[29]. The results of this study support the previous finding that CS-TPP-siRNA nanoparticles were stable, and only slight increase in particle size was observed during the experimental period of 15 days.

These results indicate that the cross-linking effect of TPP confers its properties as a stabilizer, and causes polymeric molecules to establish stronger interactions and more stable structures, which are less prone to aggregation[33]. In contrast, CS-DS-siRNA and CS-PGA-siRNA nanoparticles showed significant increase in particle size during the 15-day experimental period, which might be due to collision and adhesion of nanoparticles during storage, and consequent aggregation[36]. Moreover, CS-PGA-siRNA nanoparticles had the largest particle size after 15 days of storage, which indicated the least stable system. This result is in accordance with the previous finding that large cross-linkers poorly penetrate the CS polymer during nanoparticle formation, which leads to poor ionic interaction between the cross-linker and polymer, and thus to unstable nanoparticles that are prone to form aggregates[36]. Moreover, similar findings were observed for storage stability of siRNA-loaded CS-TPP/DS/PGA nanoparticles suspended in PBS as shown in Fig 5(B).

### 3.6 *In vitro* release study

**Fig 6:**
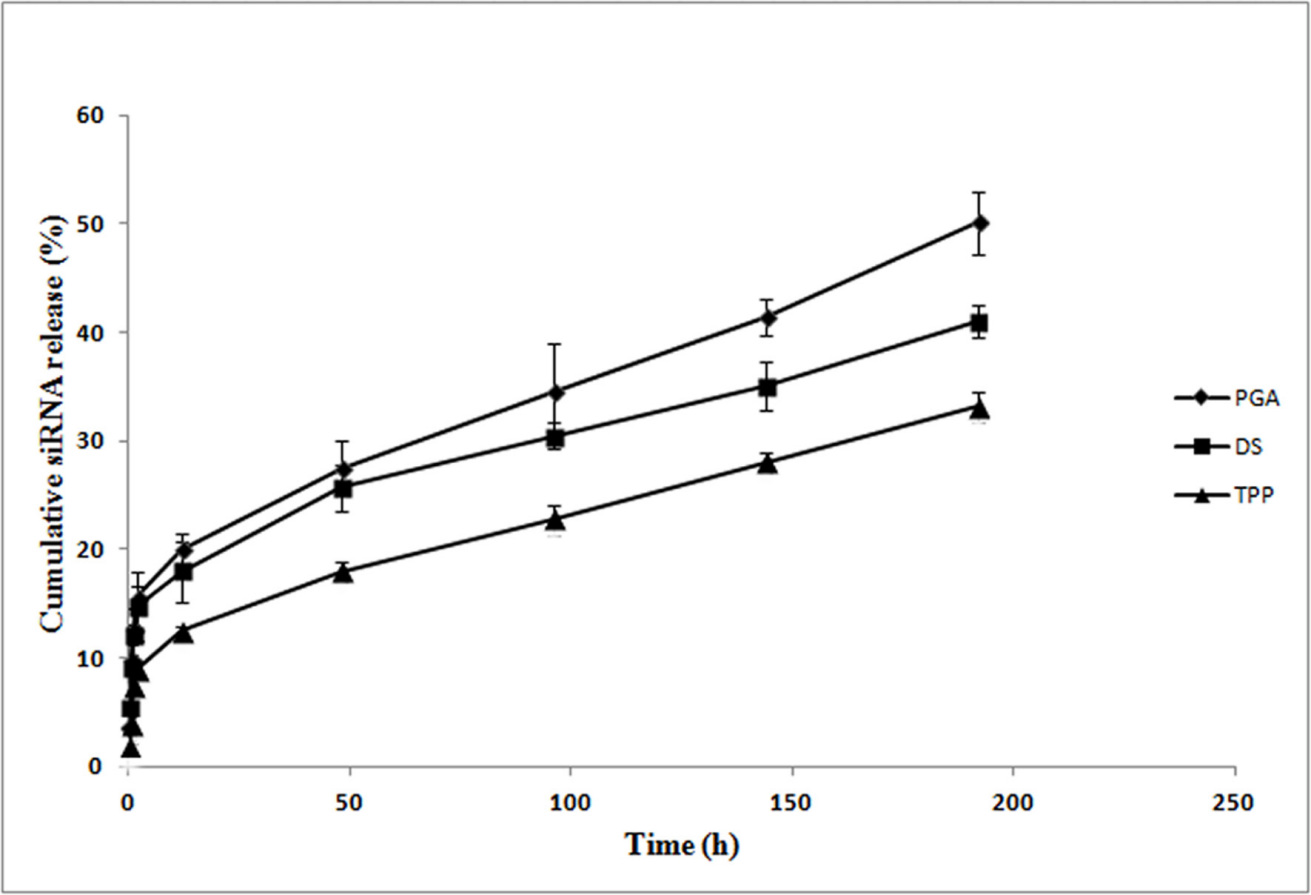
The release profile of siRNA-loaded CS-TPP/DS/PGA nanoparticles at pH 7.4, *n* = 3.

The *in vitro* release profiles of siRNA-loaded CS-TPP/DS/PGA nanoparticles were investigated for 8 days in PBS at pH 7.4, and results are shown in Fig 6. The release of siRNA was divided into 2 stages on the basis of release rate, which was calculated as the slope of the release profile. In the first stage, siRNA-loaded CS-TPP,-DS, and-PGA nanoparticles showed rapid siRNA release in the first 12 h, resulting in 12%, 18%, and 20% of cumulative release, respectively. The release of siRNA at this stage might involve the diffusion of siRNA bound near the particle surface. CS-TPP nanoparticles showed the lowest initial release of siRNA, which may be attributable to the strong cross-linking of CS-TPP-siRNA nanoparticles [37].The initial release of siRNA from CS-DS-siRNA and CS-PGA-siRNA nanoparticles was greater than from CS-TPP-siRNA nanoparticles, which was expected, due to lower binding efficiency and poor control of diffusion-based release caused by the weak cross-linking abilities of DS and PGA[37]. In the second stage, siRNA was released at a sustained constant rate from CS-TPP/DS/PGA nanoparticles for up to 8 days. TPP contributed the least cumulative siRNA release (33%) in the second stage, followed by DS (41%), and PGA (55%). This relationship was probably due to strong ionic interactions between siRNA and CS in the presence of TPP[36]. In contrast, greater release of siRNA was observed from nanoparticles cross-linked with PGA and DS, which produced a lesser degree of cross-linking[36].

### 3.7 Cytotoxicity

**Fig 7:**
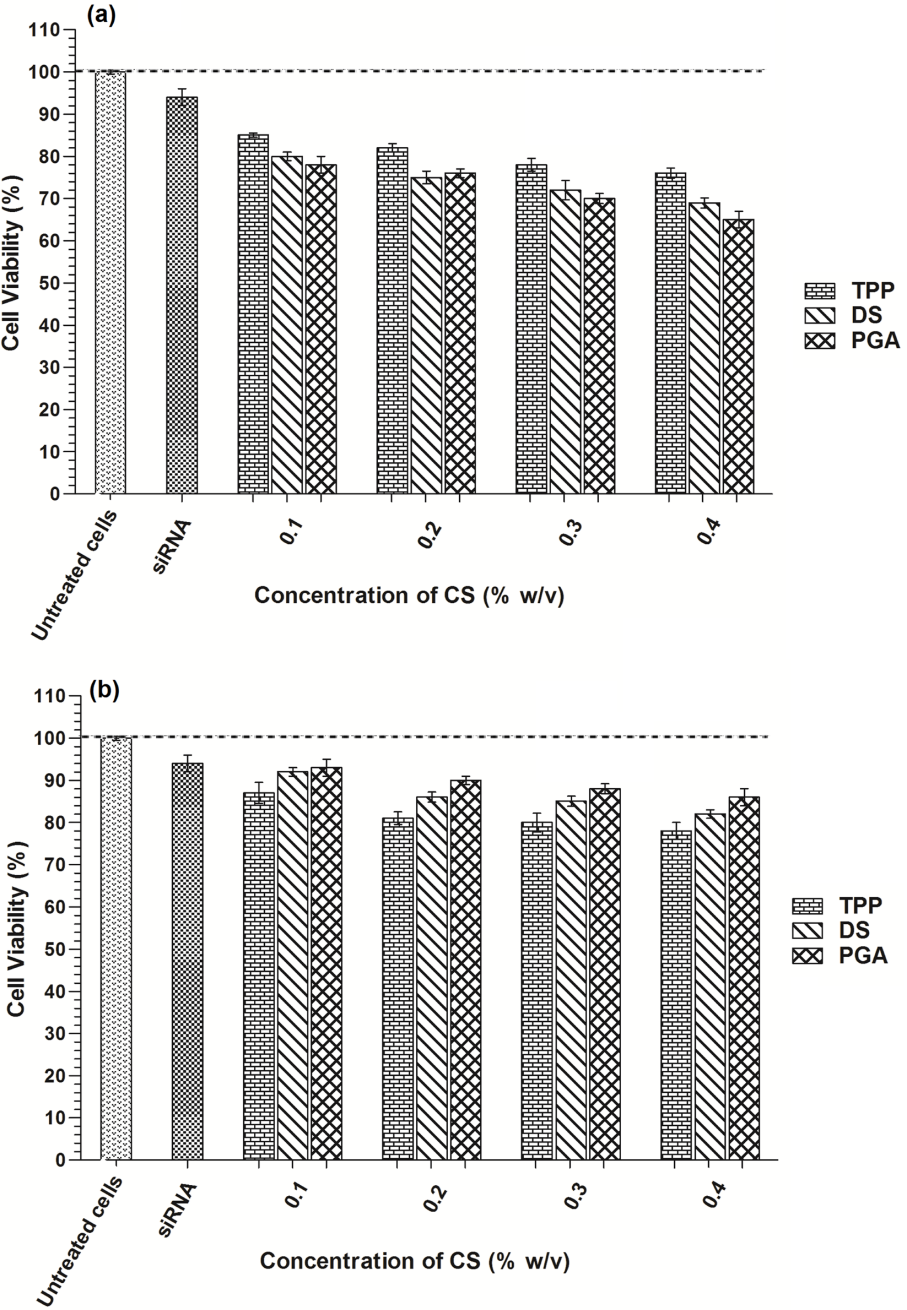
Cytotoxicity effect of siRNA-loaded CS-TPP/DS/PGA nanoparticles in DLD1 cells. After 24 h (a) and 48 h (b) incubation, *n* = 3.

To investigate the cytotoxic effect of siRNA-loaded CS-TPP/DS/PGA nanoparticles on DLD-1 cells, an alamarBluecell viability assay was performed in the presence of 10% FBS. Depending on the concentrations and cross-linkers used, all formulations caused a loss of DLD-1 cell viability after 24 and 48h incubation, as shown in Fig 7(A) and 7(B), respectively. Naked siRNA produced an 8% loss of cell viability. Generally, cell viability decreased as CS concentration increased from 0.1% to 0.4% w/v (Fig 7(A) and 7(B)).

**Fig 8:**
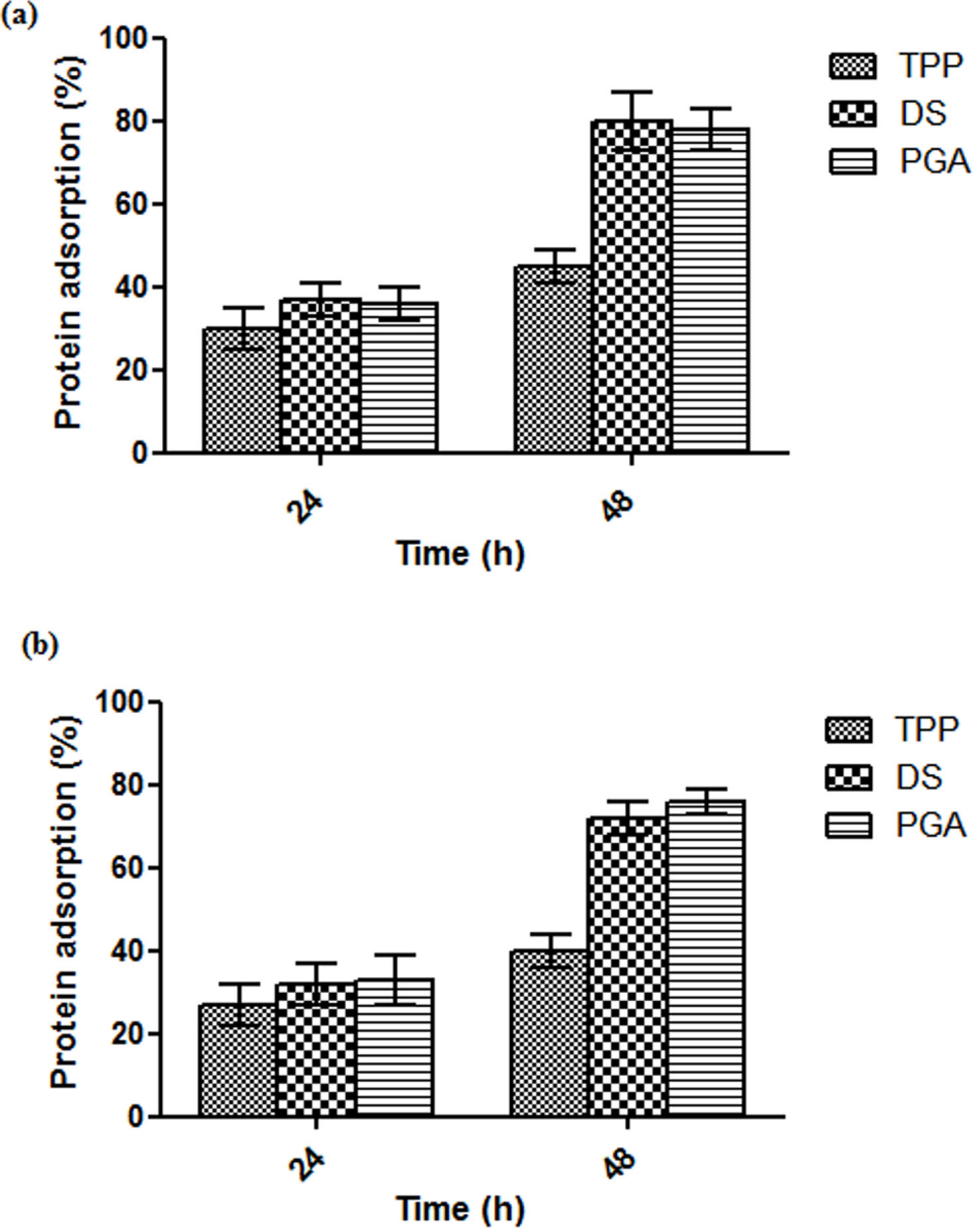
Protein adsorption ability of unloaded (a) and siRNA loaded CS-TPP/DS/PGA nanoparticles (0.1% w/v CS) in RPMI medium containing 10% FBS after 24 h (a) and 48 h (b) incubation, *n* = 3.

A 15–25% loss in cell viability was observed for CS-TPP-siRNA nanoparticles in comparison to untreated cells. Cell viability losses of 8–30% and 7–35% were observed for CS-DS-siRNA and CS-PGA-siRNA nanoparticles, respectively, depending on the concentrations used. The loss of cell viability after 24 h incubation was possibly due to initial interaction of siRNA loaded CS-TPP/DS/PGA with the cells (Fig 7(A)). Higher positive zeta potential of CS-DS- and CS-PGA-siRNA nanoparticles possibly resulted in a stronger interaction with the negatively charged cell membrane, leading to greater loss of cell viability. However, after 48 h incubation, CS-DS-siRNA and CS-PGA-siRNA nanoparticles showed significant increase in cell viability while loss of cell viability was observed for CS-TPP-siRNA nanoparticles (Fig 7(B)).This could be explained by the fact that cell viablity is also influenced by the degree of protein adsorption on the particles’ surfaces. No significant difference in protein adsorption was observed between unloaded and loaded CS-TPP/DS/PGA nanoparticles as shown in Fig 8(A) and 8(B) after 24 h incubation. However, increased protein adsorption was observed after 48 h incubation for unloaded and siRNA-loaded CS-DS and CS-PGA nanoparticles. In comparison to CS-TPP-siRNA, CS-DS- and CS-PGA-siRNA were found to adsorp more proteins on their surface after 48 h incubation in FBS and therefore, protect the cells from direct interaction with the nanoparticles. This phenomenon led to decreased cytotoxicity as previously demonstrated [38,39]. In contrast, CS-TPP-siRNA showed lower protein adsorption and resulted in loss of cell viability after 48 h incubation. However, further investigation is needed to determine this effect on the cells.

#### 3.7.1 LIVE/DEAD cell viability assay

**Fig 9:**
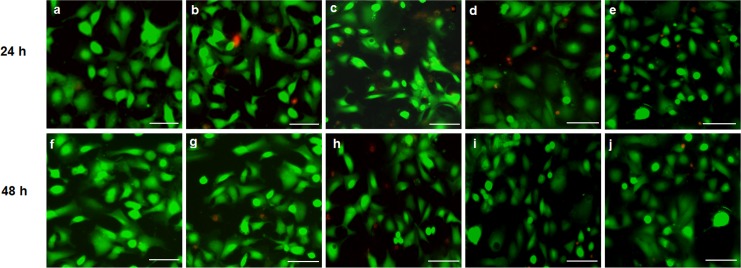
Live/dead cell viability assay of siRNA-loaded CS-TPP/DS/PGA nanoparticles (CS concentration of 0.1% w/v), in DLD-1 cells after 24 h and 48 h incubation. Untreated cells (a, f), naked siRNA (b, g), CS-TPP-siRNA (c, h), CS-DS-siRNA (d, i) CS-PGA-siRNA (e, j) after 24 h and 48 h incubation, respectively (green and red color represent viable and dead cells, respectively). Scale bar represents 10 μm.

Untreated cells did not produce any loss of cell viability while some loss was observed for the cells treated with siRNA in the presence of 10% FBS, as shown in Fig 9(A) and 9(B). These results are in accordance with cytotoxicity assays that showed increased cell death (red color) after 24 h in cells treated with CS-DS-siRNA and CS-PGA siRNA (Fig 9 (D) and 9 (E), respectively),but recovery from this effect after 48 h incubation (Fig 9(I) and 9(J)). However, CS-TPP-siRNA showed increase cell death (red color) after 48 h in cells (Fig 9(H)) in comparison to 24 h incubation (Fig 9(C)).

### 3.8 siRNA internalization/cellular uptake

**Fig 10:**
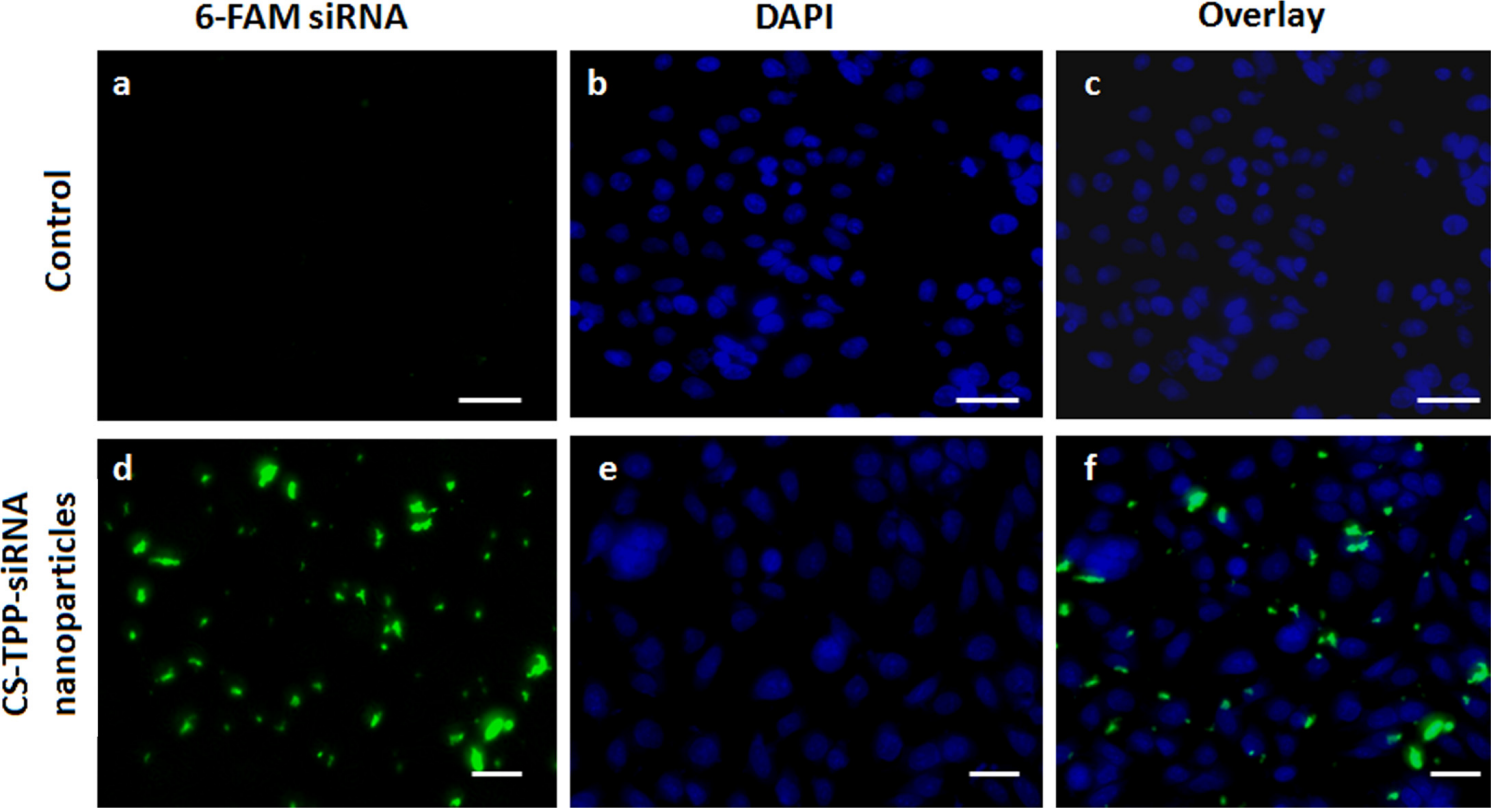
Internalization and localization of 6-FAM labelled siRNA-loaded CS-TPP nanoparticles in DLD-1 cells after 4 h incubation. Control: (a) naked 6-FAM labelled siRNA, (b) nuclear staining with Hoechst 33342 (blue), (c) an overlay of (a)-(b). Nanoparticles: (d) CS-TPP nanoparticles loaded with 6-FAM labelled siRNA (green), (e) nuclear staining with Hoechst 33342 (blue), (f) an overlay of (d)-(e). Scale bar represents 10μm.

This study was performed to determine whether CS-TPP nanoparticles facilitated the delivery of siRNA into DLD-1 cells. Fluorescein-labelled siRNA (6-FAM-siRNA) was used in this study. 6-FAM-siRNA-loaded CS-TPP nanoparticles were incubated with DLD-1 cells for 4h in the presence of 10% FBS. CS-TPP-siRNA nanoparticles infiltrated cells and were primarily distributed in the cytoplasm, which is in agreement with previous observations[40] (Fig 10D–10F). However, naked 6-FAM-siRNA was not detected (Fig 10A–10C). Fluorescence from 6-FAM-siRNA-loaded CS-TPP nanoparticles was detected in the cytoplasm suggesting that siRNA was dissociated from CS-TPP nanoparticles.

Two factors may contribute to the dissociation of the siRNA from the nanoparticles. First, the stability of nanoparticles might be affected by dilution in cytoplasm, resulting in the dissociation of nanoparticles, which subsequently cause the release of siRNA. Secondly, CS can be degraded in the presence of enzymes, resulting in deprotonation and loss of siRNA binding ability, and thus siRNA release into the cytoplasm[40].

## Conclusions

CS nanoparticles loaded with siRNA were successfully prepared by ionic gelation method susing three cross-linkers: TPP, DS, and PGA. TPP produced the smallest particle size, with high entrapment and binding efficiencies. Of the cross-linkers studied, TPP created the most stable system, leading to slow burst release of siRNA. Cytotoxic effects were determined to be dependent on concentration of CS used to prepare nanoparticles because it affects particle size and surface charge. Cellular uptake studies confirmed the successful delivery of siRNA into the cytoplasm from siRNA-loaded CS-TPP nanoparticles. The CS-TPP nanoparticles with high binding affinity for siRNA are expected could provide the ideal balance between sufficient protection and efficient intracellular release of siRNA that subsequently could produce remarkable *in vivo* antitumor effects. Based on the findings, siRNA-loaded CS-TPP nanoparticles demonstrate a great potential for clinical applications in siRNA-based cancer therapies.

## References

[1] Akhtar S, Benter IF. Nonviral delivery of synthetic siRNAs in vivo. J Clin Invest. 2007; 117:3623–32. 18060020 10.1172/JCI33494 PMC2096447

[2] De Fougerolles A, Vornlocher HP, Maraganore J, Lieberman J. Interfering with disease: a progress report on siRNA-based therapeutics. Nat Rev Drug Discov. 2007; 6:443–53. 17541417 10.1038/nrd2310 PMC7098199

[3] Kirchhoff F. Silencing HIV-1 in vivo. Cell. 2008;34:566–8. 10.1016/j.cell.2008.08.004 18724929

[4] Shim MS, Kwon YJ. Efficient and targeted delivery of siRNA in vivo. FEBS J. 2010; 277:4814–27. 10.1111/j.1742-4658.2010.07904.x 21078116

[5] Piao L, Li H, Teng L, Yung BC, Sugmito Y, Brueggemeier RW, et al Human serum albumin-coated lipid nanoparticles for delivery of siRNA to breast cancer. Nanomed-Nanotechnol. 2013;9:122–129. 10.1016/j.nano.2012.03.008 PMC3605725 22542825

[6] Lundstrom K. Latest development in viral vectors for genetherapy. Trends Biotechnol. 2003; 21:117–122. 12628368 10.1016/S0167-7799(02)00042-2

[7] Li SD, Huang L. Non-viral is superior to viral gene delivery. J Control Release. 2007; 123:181–183. 17935817 10.1016/j.jconrel.2007.09.004

[8] Kim SH, Mok H, Jeong JH, Kim SW, Park TG. Comparative evaluation of target specific GFP gene silencing efficiencies for antisense ODN, synthetic siRNA, and siRNA plasmid complexed with PEI-PEG-FOL conjugate. Bioconjugate Chem. 2006;17:241–244. 16417275 10.1021/bc050289f

[9] Kleemann E, Neu M, Jekel N, Fink L, Schmehl T, Gessler T, et al Nano-carriers for DNA delivery to the lung based upon a TAT-derived peptide covalently coupled to PEG-PEI. J Control Release. 2005;109:299–316. 16298009 10.1016/j.jconrel.2005.09.036

[10] Felgner JH, Kumar R, Sridhar CN, Wheeler CJ, Tsai YJ, Border R, et al Enhanced gene delivery and mechanism studies with a novel series of cationic lipid formulations. J Biol Chem. 1994;269:2550–2561. 8300583

[11] Liu F, Huang L. Development of non-viral vectors for systemic gene delivery. J Control Release. 2002;78:259–266. 11772466 10.1016/s0168-3659(01)00494-1

[12] Dash M, Chiellini F, Ottenbrite RM. Chiellni E. Chitosan—A versatile semi-synthetic polymer in biomedical applications. Prog Polym Sci. 2011;36:981–1014.

[13] Hamidi M, Azadi A, Rafiei P. Hydrogel nanoparticles in drug delivery. Adv Drug Deliv Rev. 2008;60:1638–1649. 10.1016/j.addr.2008.08.002 18840488

[14] Liu Z, Jiao Y, Wang Y, Zhou C, Zhang Z. Polysaccharides-based nanoparticles as drug delivery systems. Adv Drug Deliv Rev. 2008;60:1650–1662. 10.1016/j.addr.2008.09.001 18848591

[15] Illum L. Chitosan and its use as a pharmaceutical excipient. Pharm. Res. 1998;15:1326–1331. 9755881 10.1023/a:1011929016601

[16] Malmo J, Vårum KM, Strand SP. Effect of chitosan chain architecture on gene delivery: comparison of self-branched and linear chitosans. Biomacromolecules. 2011; 12:721–729. 10.1021/bm1013525 21294570

[17] Strand SP, Issa MM, Christensen BE, Varum KM, Artursson P. Tailoring of chitosans for gene delivery: novel self-branched glycosylated chitosan oligomers with improved functional properties. Biomacromolecules. 2008;9:3268–76. 10.1021/bm800832u 18834173

[18] Germershaus O, Mao S, Sitterberg J, Bakowsky U, Kissel T. Gene delivery using chitosan, trimethyl chitosan or polyethylenglycol-graft-trimethyl chitosan block copolymers: establishment of structure-activity relationships in vitro. J Control Release. 2008;125:145–154. 18023906 10.1016/j.jconrel.2007.10.013

[19] Baldrick P. The safety of chitosan as a pharmaceutical excipient. Regul Toxicol Pharmacol. 2010;56:290–299. 10.1016/j.yrtph.2009.09.015 19788905

[20] Ko JA, Park HJ, Hwang SJ, Park JB, Lee JS. Preparation and characterization of chitosan microparticles intended for controlled drug delivery. Int J Pharm. 2002;249:165–174. 12433445 10.1016/s0378-5173(02)00487-8

[21] Avadi MR, Sadeghi AM, Mohammadpour N, Abedin S, Atyabi F, Dinavand R, et al Preparation and characterization of insulin nanoparticles using chitosan and Arabic gum with ionic gelation method. Nanomed-Nanotechnol. 2010;6:58–63. 10.1016/j.nano.2009.04.007 19447202

[22] Anitha A, Deepagan VG, Rani VVD, Menon D, Nair SV, Jayakumar R. Preparation,characterisation, in vitro drug release and biological studies of curcumin loaded dextransulphate-chitosan nanoparticles. Carbohyd Polym. 2011;84:1158–64.

[23] Tsao CT, Chang CH, Lin YY, Wu MF, Wang JL, Young TH, et al Evaluation of chitosan/γ-ply(glutamic acid) polyelectrolyte complex for wound dressing materials.Carbohyd Polym. 2011;84:812–819.

[24] Lin YH, Chung CK, Chen CT, Liang HF, Chen SC, Sung HW. Preparation of nanoparticles composed of chitosan/ poly-gamma-glutamic acid and evaluation of their permeability through caco2 cells. Biomacromolecules. 2005;6:1104–12. 15762683 10.1021/bm049312a

[25] Papadimitriou AS, Achilias DA, Bikiaris DN. Chitosan-g-PEG nanoparticles ionically crosslinked with poly(glutamic acid) and tripolyphosphate as protein delivery systems. Int J Pharm. 2012;430:318–327. 10.1016/j.ijpharm.2012.04.004 22521711

[26] Calvo P, L´opez CR, Vila-Jato JL, Alonso MJ. Novel hydrophilic chitosan- polyethylene oxide nanoparticles as protein carriers. J Appl Polym Sci. 1997;63:125–132.

[27] Fan W, Yan W, Xu Z, Ni H. Formation mechanism of monodisperse, low molecular weight chitosan nanoparticles by ionic gelation technique. Colloids Surf B. 2012; 90:21–27. 10.1016/j.colsurfb.2011.09.042 22014934

[28] Katas H, Alpar HO. Development and characterisation of chitosan nanoparticles for siRNA delivery. J Control Release. 2006;115:216–225. 16959358 10.1016/j.jconrel.2006.07.021

[29] Rampino A, Borgogna M, Blasi P, Bellich B, Cesaro A. Chitosan nanoparticles: Preparation, size evolution and stability. Int J Pharm. 2013;455:219–228. 10.1016/j.ijpharm.2013.07.034 23886649

[30] Csaba N, Koping-Hoggard M, Alonso MJ. Ionically crosslinked chitosan/ tripolyphosphate nanoparticles for oligonucleotide and plasmid DNA delivery. Int J Pharm. 2009;382:205–214. 10.1016/j.ijpharm.2009.07.028 19660537

[31] Vanderberg GW, Drolet C, Scott SL, de la Noue J. Factors affecting protein release from Alginate chitosan coacervate microcapsules during production and gastro/intestinal simulation. J Control Release. 2001;77:297–307. 11733097 10.1016/s0168-3659(01)00517-x

[32] Hickerson RP, Vlassov AV, Wang Q, Leake D, llves D, Gonzalez-Gonzalez E, et al Stability study of unmodified siRNA and relevance to clinical use. Oligonucleotides.2008;18:345–354. 10.1089/oli.2008.0149 18844576 PMC2829675

[33] Rodrigues S, Costa AMR, Grenha A. Chitosan/ carrageenan nanoparticles:effect of Cross linking with tripolyphosphate and charge ratios. Carbohyd Polym. 2012;89:282–289 10.1016/j.carbpol.2012.03.010 24750635

[34] Raja MAG, Katas H, Hamid ZA, Razali NA. Physicochemical properties and in vitro cytotoxicity studies of chitosan as a potential carrier for Dicer-substrate siRNA. J Nanomater. 2013; 10.1155/2013/653892

[35] Al-Qadi S, Grenha A, Remuñán-López C. Microspheres loaded with polysaccharide nanoparticles for pulmonary delivery: Preparation, structure and surface analysis Carbohyd Polym. 2011;86: 25–34.

[36] Tsai ML, Chen RH, Bai SW, Chen WY. The storage stability of chitosan/tripolyphosphate nanoparticles in a phosphate buffer. Carbohyd Polym. 2011;84:756–761.

[37] Dudhani AR, Kosaraju SL. Bioadhesive chitosan nanoparticles: Preparation And characterization. Carbohyd Polym. 2010;81:243–251.

[38] Hu W, Peng C, Lv M, Li X, Zhang Y, Chen N, et al Protein corona-mediated mitigation of cytotoxicity of graphene oxide. ACS NANO. 2011;5:3693–3700. 10.1021/nn200021j 21500856

[39] Ge C, Du J, Zhao L, Wang L, Liu Y, Li D, et al Binding of blood proteins to carbon nanotubes reduces cytotoxicity. PNAS. 2011;108:16968–73. 10.1073/pnas.1105270108 21969544 PMC3193254

[40] Savic R, Luo LB, Eisenberg A, Maysinger D. Micellar nanocontainers distribute to defined cytoplasmic organelles. Science. 2003;300:615–8. 12714738 10.1126/science.1078192

